# Evaluating Federated Learning Simulators: A Comparative Analysis of Horizontal and Vertical Approaches

**DOI:** 10.3390/s24165149

**Published:** 2024-08-09

**Authors:** Ismail M. Elshair, Tariq Jamil Saifullah Khanzada, Muhammad Farrukh Shahid, Shahbaz Siddiqui

**Affiliations:** 1 Information Systems Department, Faculty of Computing and Information Technology, King Abdulaziz University, Jeddah 21589, Saudi Arabia; tkhanzada@kau.edu.sa; 2 Computer Systems Engineering Department, Mehran University of Engineering and Technology, Jamshoro 76062, Pakistan; 3 Department of Computer Science, National University of Computer and Emerging Sciences, Karachi 75030, Pakistan; mfarrukh.shahid@nu.edu.pk (M.F.S.); shahbaz.siddiqui@nu.edu.pk (S.S.)

**Keywords:** federated learning, federated learning simulators, federated learning topology, vertical and horizontal federated learning

## Abstract

Federated learning (FL) is a decentralized machine learning approach whereby each device is allowed to train local models, eliminating the requirement for centralized data collecting and ensuring data privacy. Unlike typical typical centralized machine learning, collaborative model training in FL involves aggregating updates from various devices without sending raw data. This ensures data privacy and security while collecting a collective learning from distributed data sources. These devices in FL models exhibit high efficacy in terms of privacy protection, scalability, and robustness, which is contingent upon the success of communication and collaboration. This paper explore the various topologies of both decentralized or centralized in the context of FL. In this respect, we investigated and explored in detail the evaluation of four widly used end-to-end FL frameworks: FedML, Flower, Flute, and PySyft. We specifically focused on vertical and horizontal FL systems using a logistic regression model that aggregated by the FedAvg algorithm. specifically, we conducted experiments on two images datasets, MNIST and Fashion-MNIST, to evaluate their efficiency and performance. Our paper provides initial findings on how to effectively combine horizontal and vertical solutions to address common difficulties, such as managing model synchronization and communication overhead. Our research indicates the trade-offs that exist in the performance of several simulation frameworks for federated learning.

## 1. Introduction

Federated Learning (FL) was introduced in 2016 by McMahan et al. [1] as method to enhance conventional machine learning (ML) techniques. In ML, the accuracy of trained models depends on several factors, including data size and quality of data, as well as whether the data are Identical and Independently Distributed (IID) or not. Before the introduction of FL, achieving a model with high accuracy required a large dataset, which can only be obtained by collecting data from various distributed sources such as servers, organizations, mobile devices, and others. However, this practice leads to exchanging data, which results in compromising data privacy [2]. In addition, some concerns were raised in sharing data along with the safety of data, and the huge size of data required to be moved to a central server in order to produce highly accurate models [1]. According to [3], the total number of cases in American courts that involving data breaches in the healthcare industry rose from 16 in 2005 to 344 in 2022. In addition, according to the prediction made by [4] the global data volume is projected to exceeds 180 zettabytes by 2025. Nevertheless, in order to enhance the accuracy of predictions made by trained models, it is necessary to distribute this vast amount of data beyond the organization’s local servers and store it on a centralized platform. Therefore, the FL model has tackled these challenges by training datasets from several resources and organizations in a decentralized way without the need to transfer data to a central server. This is in contrast to traditional machine learning FL has overcome the challenges of security, safety, regulations, and data volume, ensuring that all data remains in the original source. Subsequently, every node generates a local model that will be combined with other models using an algorithm such as FedAvg [1], resulting in a global model on a server. Lastly, the global model is distributed to all nodes and the process is then repeated. The source of data in FL could vary from clients’ mobile devices, organizations’ servers, wearable devices, the Internet of Things (IoTs), and all kind of devices generating private data from different resources [5], which can be trained without sharing data. Bonawitz et al. [6] introduced an implementation considered as one of first FL implementations using mobile devices with a centralized approach where the devices are the ones to train and process their own local data and share the new generated model with the server. After receiving new models, the server makes a better global model by aggregating the weights from massive devices. In a bid to realize its underlying technology in practice, FL is always undergoing constant advancement, where an array of approaches and techniques are employed. Furthermore, FL can be characterized in several ways based on the topology, algorithm, and the implementation domain. Recently, the term Vertical Federated Learning (VFL) was introduced by [7] to solve the challenge in distributing data vertically and across multiple devices, without compromising data privacy. VFL is applied in cases where the clients share the same sample space while not matching in the feature space [8]. Various simulation frameworks have been developed in the field of FL to facilitate and ease the process of implementing new models, which are not widely recognized such as FedML [8], and FLSim [9]. Mohamed et al. [10] proposed a novel approach for detecting attacks using vehicular edge computing (VEC) and suggested a Federated Deep Learning-based Intrusion Detection System (FED-IDS) to make smart transportation systems (STSs) safer by spreading the learning process to vehicular edge nodes and then using blockchain to verify the local updates from the connected vehicles, therefore preventing vehicles from sending updates that are not reliable. The study utilized two datasets: Car-Hacking and TON IoT. The researchers identified the security vulnerabilities in STSs, particularly cyber-threats to vehicular networks, emphasized the importance of intrusion detection systems (IDSs), and highlighted the challenges associated with centralized learning. Whitworth et al. [11] introduced a hybrid approach for the detection of DDos attacks in 5G-enabled airports using a multi-channel CNN-GRU methodology. Using Gramian Angular Fields (GAFs) for transforming the time-based features into 2D texture images, a parallel architecture analyzes N time series image features concurrently. The suggested methodology consists of four sequential steps: data collection, feature extraction, GAF conversion, and CNN analysis while incorporating a gated recurrent unit (GRU) for classification. The model accuracy is 98.6% for the Cranfield embedded Systems Attack Dataset and, on the CIC2019 DDoS Dataset, it attained an accuracy of 89.08%. However, after deploying the feature optimization, the model achieved an accuracy of 98.36%. Nickolaos Koroniotis et al. [12] provides a comprehensive analysis for applications, architecture, threats, and security measures associated with the incorporation of IoT in smart airports. They discuss the benefits of IoT in airport services, while providing a concise overview of the vulnerabilities that comes along with the benefits. This study encompasses a range of topics from the analysis of applications to proposing solutions for enhancing the passenger experience of the smart airports by automating several processes. In the comparative analysis of FL frameworks, Wu et al. [13] conducted a comparison examination of FL frameworks, specifically comparing Flower, LEAF, Syft, and FedScale. Their research revealed that Flower over-performed the other frameworks in terms of both communication-agnostic and language-agnostic capabilities. These characteristics enable researchers to implement FL models using range of languages. In addition, Flower demonstrated exceptional scalability, with the ability to effectively handle a vast number of devices, reaching into the millions. Hipolito et al. [14] conducted another study to compare FedML, Flower, and Flute frameworks. The study demonstrated that Flower lacks support for both cloud integration and multi-GPU environments, whereas Flute and FedML do provide such support. Furthermore, Flute was the lone platform that support performance optimizations. Notably, each of these papers conducted a comparison of multiple frameworks and case studies. Our research is distinctive since it encompasses both VFL and HFL, areas which were not addressed in the previous studies. Our Analyzed involved evaluating various simulators and conducted a comparative study of four of these simulators. We utilize two different datasets to identify the optimal scenarios and measure the variation in accuracy. Table 1 shows the summary of existing work along with its solutions and limitations.

### 1.1. Motivation

FL distributes the power of machine learning among devices, enabling local model training rather than depending on a central server. This approach preserves privacy and confidentiality by not releasing raw data while still facilitating collaborative learning. Each device gathers information from its surroundings and provides updates to a central server, which integrates these updates to enhance the overall model. Clear and effective communication with cooperation between remote devices help protect data privacy, ensure scalability, and improve the robustness of FL models through the network architecture. The main challenges include managing communication and ensuring both model accuracy and synchronization across multiple devices. FL offers possibilities for the creation of traffic prediction models in applications for smart cities. Data from infrastructure sensors and vehicles throughout the city would be gathered and used to train local models. These local models would then be shared with a central server to create a comprehensive traffic forecasting system. This would maintain the privacy of individual data while enabling more effective traffic control and planning. These use-cases highlight the motivating factors for the study covered in this paper. By reviewing different FL frameworks that work on both HFL and VFL approaches, this research aims to evaluate these frameworks. Through an analysis of the latest implementations of FL, network topologies, and a comparison of decentralized and centralized frameworks, this paper aims to provide insightful analysis for the development and implementation of FL systems in simulation frameworks.

### 1.2. Contributions

Based on the motivation, the three main contributions of our research are listed as follows:

1. This paper analyzes the advantages and drawbacks of decentralized and centralized FL topologies, identifying key enhancements and data distribution strategies applicable to different scenarios.
2. We evaluate various FL frameworks, including Flower, Flute, FedML, and PySyft, across HFL and VFL approaches, using logistic regression with the FedAvg algorithm.
3. Our study provides valuable insights for researchers and practitioners to identify suitable simulation frameworks for validating new algorithms and implementations in FL.

## 2. Comprehensive Study of FL

In this section, we explore the topology in FL, emphasizing the underlying simulation frameworks and platforms that enable these architectures. We discuss several algorithms in FL while explaining how the FedAvg algorithm is distributed and communicated across VFL and HFL systems, highlighting the designs of centralized and decentralized FL that facilitate effective model training and privacy preservation.

### 2.1. Topology in FL

FL is a novel method of machine learning that involves distributing model training across numerous devices or nodes. This methodology ensures data privacy and security by keeping the data confined to the client devices [15]. This strategy utilizes the processing capabilities of edge devices and reduces the necessity for centralized data collection. FL can implement data distribution using two main topologies: centralized and decentralized. Every topology possesses unique operating frameworks, advantages, and difficulties that are tailored to specific application requirements and scenarios.

#### 2.1.1. Centralized FL

**Figure 1:**
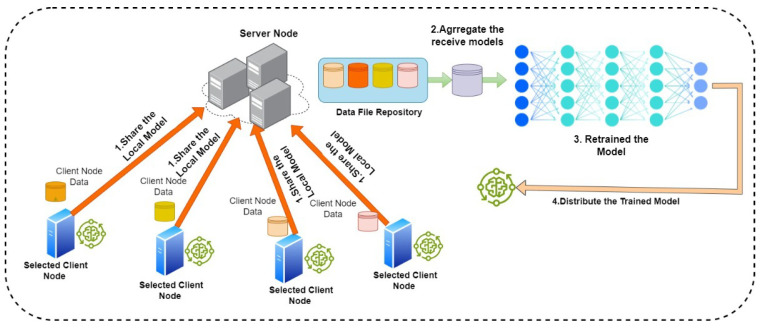
The steps to complete training cycle within classic centralized learning.

The first model in FL was developed on an aggregation server topology where all nodes train data locally then share a new model with the server, which aggregates and averages all received models in order to generate a global model. A typical centralized learning contains five steps [1] (as depicted in Figure 1) in order to complete the training cycle, which are follows:

- Client selection: the server employs an eligibility verification process for participant devices, which involves checking for the presence of specific conditions, such as being connected to an unmetered Wi-Fi network, having an idle status, and being connected to a charging source. The aim of this process is to prevent any negative impact on the user of the device. The area of battery and charging has been explored in little research. In [16], the authors presented power-aware FL using energy-aware FL (EAFL), which selects devices to maintain low time-to-accuracy while using conservative amount of power and decreases the dropping off of clients, which leads to an increase in the accuracy of models.
- Broadcast: the chosen clients retrieve the current model weights and training instructions, e.g., TensorFlow-Federated (TFF) and PySyft [17] from the server.
- Client computation: every selected device performs a local computation by executing the training instructions, which involve running the stochastic gradient descent (SGD) algorithm on the device’s local data, to generate and update the model.
- Aggregation: the server gathers a collective update of the device computations. At this point, the most commonly utilized algorithm is the FedAvg algorithm within the FL domain.
- Model update: using the update gathered from the participant clients in the current round, the server performs a local update to the shared model. However, in centralized learning, two main topologies have been used to create a federated environment, namely, the aggregation server and hierarchical learning.

#### 2.1.2. Aggregation Server Topology

The aggregation server, which is referred to as a star topology, has the most contributions within FL topologies. In the mobile sector, we find Alphabet-applied FL on several services in order to improve the next word prediction from 6.3 billion sentences in Gboard service. Another implementation is to improve the “OK Google” service, which uses voice recognition. On the other hand, Apple applied FL to improve Siri services by training models on devices, which improved the response of “Hey Siri” [15]. Furthermore, the amount of data generated today is huge [18]. Training these huge data would improve many industries and applications. Authors in [15] assume that, whenever the dataset is small and training is local, FL-generated models are clearly more accurate [19]. FL is used to optimize learning in IoT devices. In closed-circuit television (CCTV), due to security reasons where data are protected, FL is applied in organizations to have a proper training on devices locally without exchanging data. FedVision proposed, for visual object detection, to detect flames through CCTV cameras in order to provide an early alarm. The data were trained from three different organizations with more than 100 factories [20]. However, in the domain of the Internet of Everything (IoE), ref. [5] proposed a model where FL was implemented on an IoE scheme to be connected via multiple networks.In [21] introduced, in Intelligent transportation systems (ITSs), a framework for FL Attention-based Spatial-Temporal Graph Neural Networks (FASTGNNs) for traffic speed forecasting that is presented based on a Graphs Neural Network (GSS), which gathers trained models on a server then combines models using FedAvg to generate models with higher accuracy and less errors compared to other approaches in ITS, such as Spatio-Temporal Graph Convolutional Networks (STGCNs) [22], and Graph WaveNet [13]. Furthermore, in the field of the Internet of vehicles (IoV), where the new cars providing a self-drive functionality, In [23] introduced a way to improve the recognition of traffic signs in streets by using Spike Neural Networks (SNNs) with FL, where they proposed a FedSNN-NRFE algorithm that shows a higher accuracy in trained models, reaching up to 94.9%.

Moreover, another implementation introduced to enhance data trafficking within topology, ref. [24], is an Internet traffic classification protocol (FLIC) developed by focusing on data leakage with an accuracy of 88%, which considered a higher accuracy compared to centralized deep learning. In addition, FLIC provided evidence for a positive relation between data volume and accuracy. Another research was introduced by [25] using Network Simulator-3 (NS3) and the User Datagram protocol (UDP) to guarantee the reliability and efficiency within transport processes.

##### Hierarchical Topology

**Figure 2:**
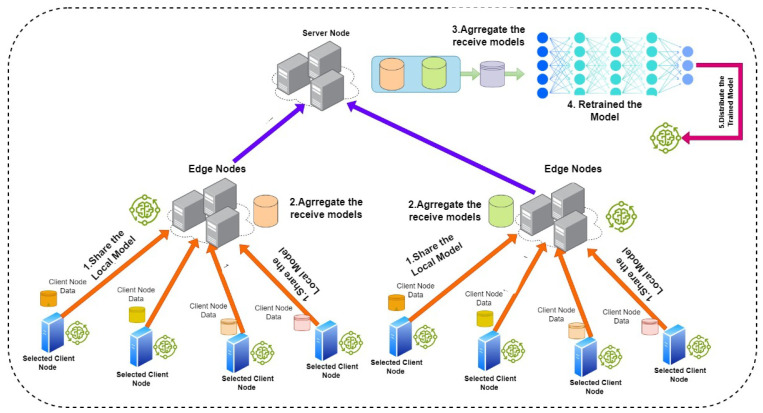
Centralized FL: hierarchical topology.

Hierarchical topology is considered as a star topology with additional layer/layers that operate between server and client nodes (as depicted in Figure 2). In hierarchical topology, the lower layer contains scattered nodes into a pool (child node). Notably, the middle layer contains edge-servers (parent), where each parent contains a group of child nodes that are gathered either randomly or based on a similarity in location or performance. In addition, the parent node represents an additional layer that could be virtual or physical nodes. The functionality of the parent node is to aggregate models from child nodes and then send it to the main server at the top layer [26]. The main idea in hierarchical topology is to reduce direct communication between the server and nodes, whereas, in some cases, an FL model contains millions of devices, which leads to high communication on a single server. After experimenting with hierarchical topology, it was found that it would outperform aggregation server topology in performance when the devices are no longer than two edge devices [27]. FL with Cluster Construction and Hierarchical Aggregation (FedCH), implemented by [28], focuses on the performance of the hierarchical platform by determining the optimal number of clusters and devices within each cluster.

#### 2.1.3. Decentralized FL

The concept behind decentralized learning is to perform the learning without having a server node that gathers and aggregates models from participant nodes. The major challenge within centralized learning is bottlenecks [29]. Therefore, a decentralized training is proposed where nodes communicate directly, exchange models, and aggregate models locally [30]. In theory, decentralization is faster than centralized topology, and decentralized algorithms are capable of decreasing the cost of communication with the servers by transferring the communication cost to be between clients [31]. Mainly, the use of decentralized FL is in cross-silos where data are located in organizations, hospitals, or institutes, and the size of data is huge. In this subsequent section, we discuss two main topologies, which are peer to peer (P2P) and ring topologies.

##### Peer-to-Peer (P2P) Topology

**Figure 3:**
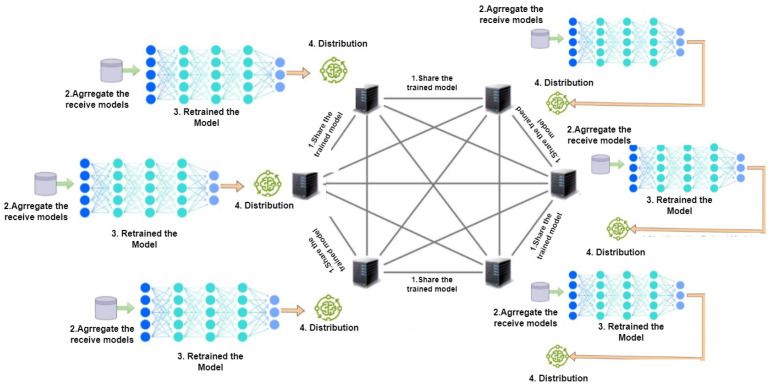
Decentralized FL: P2P topology.

The training process assumes nodes as integral to the unstructured P2P topology. Each peer is a silo that operates independently to generate a trained model (as depicted in Figure 3). Silos are capable of communicating directly among a subset of peers within the network. These peers generate models where each node works simultaneously in training data at several iterations, then exchange models with other peers to combine models locally [32]. The previous process is repeated between all nodes and none of the models generated by peers are sent to the central server after the training process. The author in [30] call this a mutual trust between neighbors, so that every peer taking part in improving the model by sharing models with the neighbors rather than with a global model concept, as silos can communicate with each other. Braintorrent [32] provided a proof of concept by applying P2P on medical centers in order to train data, and results showed better accuracy compared to central FL. However, ref. [33] introduced a solution in multi-energy system (MESs), which supports green energy by decreasing energy and carbon emission from buildings. The experiment performed on three buildings and training was performed using joint P2P energy and the carbon algorithm (Fed-JPC).

##### Ring Topology

**Figure 4:**
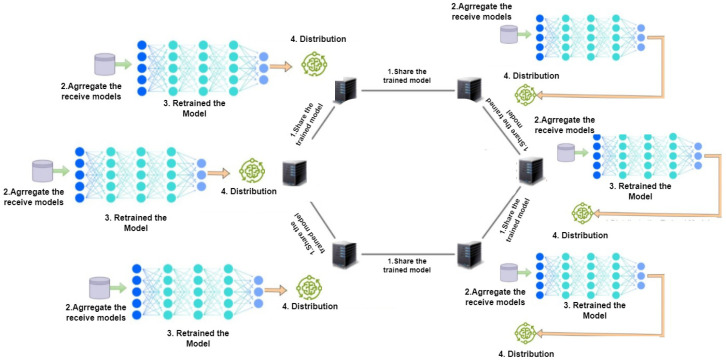
Decentralized FL: ring topology.

Ring topology is a decentralized FL where all nodes are linked to the next nodes that share models between nodes into two directions: clockwise or counterclockwise (as depicted in Figure 4). One of the challenges in ring topology is the high dependency on neighbor nodes. Wang et al. proposed [17] a ring FL organized into trusted and non-trusted nodes, where untrusted nodes can communicate only with a trusty node in order to find the malicious nodes. However, ring typology’s scalability is affected by the total number of nodes, due to the fact that training is limited to neighbor nodes, then passing a new model to the neighbor node, which requires a longer time to go through all nodes in a sequential form.

Tornado Aggregate [34] was implemented in order to increase scalability and accuracy by clustering the clients into groups, when the number of clients was high. In addition, they proposed ring chaining, where, in each iteration, the clients were grouped in a new way in order to reduce bias in the learning process.

### 2.2. FL Frameworks and Simulators

To implement an FL model, researchers have to use a library within a platform, which requires an expert within the team to implement it; in addition, it requires having a dedicated budget. Different platforms provide the capability to implement an FL solution such as PyTorch, and TensorFlow Federated (TFF). Consequently, frameworks and simulators were developed as a novel method in FL, to demonstrate and facilitate the work of researchers, allowing them to perform experiments, evaluate new ideas, and to validate new concepts. Guangsheng et al. [35] introduced the IronForge platform, which surpasses FedAvg in terms of accuracy and security. They conducted experiments between platforms using the FLSim framework, which enables the implementation of a decentralized FL. In ns3-fl [36], a new framework was developed by integrating two existing frameworks, ns3, which supports advanced network configuration, and FLSim, which facilitates the simulation of FL. This integration aims to calculate energy consumption effectively. Li et al. [37] introduced a benchmark simulation in cybersecurity that includes built-in implementations of representative attack and defense scenarios in FL. Blades offers an open-source benchmark that simulates these scenarios, making it readily available for use by other researchers. Critical characteristics of cutting-edge FL simulators have been identified through an examination of numerous research papers [38]. What follows is the discussion of these characteristics:

- Data distribution and partitioning: state-of-the-art FL simulators ought to offer adaptable mechanisms for data partitioning and distribution. This feature helps with different ways of dividing data, such as IID (Independent and Identically Distributed) and non-IID data, so that you can accurately simulate real-life situations. Researchers rely heavily on the ability to modify data partitioning and distribution in order to evaluate the performance and robustness of their FL algorithms across a wide range of data conditions.
- Communication topology: simulators should provide communication topologies to emulate diverse network architectures and the interdependencies of participants regarding trust. With support for star, tree, and mesh topologies, researchers can now evaluate the impact of communication patterns on the performance and privacy of their FL algorithms.
- Computation patterns of participants: simulators should be designed to accommodate varying resource constraints, communication dynamics, and participant computation patterns. This functionality allows scientists to evaluate the robustness and flexibility of their FL algorithms when confronted with random connectivity and diverse participants.
- Privacy and security features: FL frameworks should incorporate advanced privacy and security capabilities like secure aggregation, differential privacy, and, where necessary, training on encrypted data.
- Scalability and efficiency: FL frameworks must scale to handle large deployments and be efficient in optimizing the allocation of resources. They need to handle performance implications caused by participant heterogeneity, reduce communication overhead, and improve convergence.
- Heterogeneity and adaptability: to accurately simulate real-world deployment, simulators should have the capability to host heterogeneous hardware and software in conjunction with varying network conditions. This way, researchers can evaluate how adaptive and robust FL algorithms are in an extended environmental setup.

The value that FL platforms bring is their support for distributed machine learning tasks in heterogeneous environments and devices. These FL platforms have been developed to meet the specific requirements of an FL system and include a variety of functionalities and capabilities for data confidentiality, scalability, communication efficiency, model training, and more, all within the context of distributed learning. In FL, the following simulation frameworks and platforms considered as the state of the art.

#### 2.2.1. TensorFlow Federated (TFF)

TFF considered as one of the main platforms in implementing FL solutions where we find many implementations and examples provided. The TFF interface is structured into two layers [39]:

- FL API:
  This layer provides a suite of sophisticated high-level interfaces that enable developers to employ the provided implementations of federated training and evaluate new models.
- Federated Core API:
  This foundational layer forms the base in FL by providing lower-level interface, whereas in the heart of the system lies a collection of foundational interfaces designed to succinctly articulate innovative federated algorithms. These interfaces enable the fusion of TensorFlow with distributed communication operators.

#### 2.2.2. PySyft

- PySyft is an open-source library for encrypted and privacy-preserving machine learning comprising various deep learning frameworks in order to carry out computations that are secure and private [40]. Federated averaging in PySyft supports a VFL, making it possible to achieve privacy-preserving collaboration among powerful entities with different feature sets. PySyft is considered as a suitable framework for such sensitive applications where strict privacy guarantees are required.

#### 2.2.3. Flower

The Flower framework operates and is licensed under Apache 2.0 to facilitate the implementation and experimentation of FL algorithms and models [13]. Flower was introduced, mainly, to increase the total number of devices on FL platforms to 15 million real clients, which are capable of training and evaluating the generated models [41]. Flower has allowed FL researchers to apply concepts and research in real systems using mobile devices, computers, and the cloud.

#### 2.2.4. FL Utilities and Tools for Experimentation (Flute)

In experiments, Flute is an open-source FL framework that was built on the PyTorch platform for research and off-line simulations [14]. Flute is capable of enhancing the implementation of experiments in both the privacy and scalability of FL. In addition, it provides default support to the horizontal communication in which the feature space is shared between the clients, but the samples are different. Flute is specifically useful for researchers and developers in need of a special toolset to easily afford support for their FL experiments while providing a set of algorithms, such as FedAvg, DGA [14], and FedAdam [42], which come with a significantly scalable platform.

#### 2.2.5. FL Simulator (FLSim)

FLSim is a flexible simulation framework, which consists of a set of components as an open library of building blocks to simulate FL settings. FLsim is capable of integrating with other systems and platforms through the collection of APIs [9].

#### 2.2.6. Federated Machine Learning (FedML)

FedML is a research library and benchmark introduced to facilitate the development of new algorithms in FL and performing comparisons [8]. At its core, FedML features two API layers: FedML-API (high level) and FedML-core (low level). The design of FedML-core strategically separates communication and model training tasks. Concurrently, FedML-API provides robust support for security- and privacy-related algorithms, enhancing the framework’s utility for advanced FL research. Concerning FedAvg, FedML supports VFL, where clients have different feature spaces but share the same sample space.

### 2.3. FL Algorithms for Model Aggregation

In FL, after training the model on the local device, the server receives new local models to update the global model in an aggregation process. The most common algorithms for model aggregation in FL are as follows:

- Federated Average (FedAvg): considered as the first algorithm in FL [1] and the most commonly used algorithm, which is a method that combines updates from individual clients to create a new global model by calculating the average of their contributions.
- Hierarchical Federated Averaging (HierFAVG): an algorithm that applies the concept of hierarchical FL on a multi-layer framework, where the models are shared from clients with an edge node that is in the middle between clients and the server [26].
- Federated Matched Averaging (FedMA): introduced by [43], which allows convolutional neural networks (CNNs) [44] to adapt to the heterogeneity of the datasets by averaging and matching elements in a layer-wise manner. One of the advantages in FedMA is the ability to run with less communication rounds in FL.
- FedProx is a modified version of FedAvg that incorporates a proximal term to specifically deal with the differences among clients, hence enhancing the management of non-IID data. The FedProx algorithm improved the accuracy in highly heterogeneous settings of FL [45].

The goal of these algorithms is to improve efficiency, robustness, and precision in relation to the heterogeneity of data, communication constraints, and rates of convergence, among other things. In this research work, we examine the FedAvg algorithm with logistic regression, taking into account HFL and VFL approaches.

#### 2.3.1. FedAvg in HFL

Horizontal FL involves the aggregation of data from various clients or devices that are of the same type but originate from separate sources [46]. Clients divide the data into horizontal partitions, each containing a subset of the features or attributes. We can express Equation (1) for HFL as follows:

(1) $${\mathrm{Model}}_{\mathrm{Global}}=\frac{1}{N}\sum_{i=1}^{N}{\mathrm{Model}}_{{\mathrm{Local}}_{i}}$$

where

- ${\mathrm{Model}}_{\mathrm{Global}}$ represents the global model trained across all clients.
- ${\mathrm{Model}}_{{\mathrm{Local}}_{i}}$ represents the local model trained on client *i*.
- *N* represents the total number of clients participating in FL.

Algorithm 1 enables the implementation of HFL by coordinating the training of local models on clients and aggregating these local models to generate the global model. Computed as a weighted average of the local models, the global model corresponds to the idea of HFL in which the global model is an average of models trained on data partitions from several clients [47], as shown in Equation (2):

(2) $$G_{t}\leftarrow \sum_{c=1}^{m}\frac{d_{c}}{d}L_{c}$$

where $d_{c}$ is the number of data points on client *c* and *d* is the total number of data points across all selected clients. Locally, each client updates its model by replacing $L_{c}$ with $G_{t}$ and then training for *E* epochs. For each mini-batch *b* within an epoch, the client updates its model parameters using gradient descent [1], with a learning rate η as shown in Equation (3):

(3) $$L_{c}\leftarrow L_{c}-\eta \nabla L\left(L_{c},b\right)$$

**Algorithm 1** FedAvg with HFL.**Require:** Total clients *K*, batch size *B*, rounds *T*, epochs *E*, learning rate η  1:**Server:**  2:Initialize global model $G_{0}$  3:**for** each round t=1, 2, …, T **do**  4:     Select m=C×K clients  5:     **for** each client *c* in selected clients **do**  6:          Send global model $G_{t}$ to client *c*  7:          Client *c* trains locally and sends updated model $L_{c}$ to server  8:     **end for**  9:     Aggregate updates to update global model:10:     $G_{t}\leftarrow \sum_{c=1}^{m}\frac{d_{c}}{d}L_{c}$11:**end for**12:**Client *c* update:**13:Set local model $L_{c}\leftarrow G_{t}$14:**for** epoch e=1 to *E* **do**15:     **for** batch b=1 to *B* **do**16:          $L_{c}\leftarrow L_{c}-\eta \nabla L\left(L_{c},b\right)$17:     **end for**18:**end for**19:Return $L_{c}$ to server

This iterative method improves the global model by using distributed training and various client data without sacrificing privacy. Starting with the server establishing the global model, $G_{0}$, the FedAvg process proceeds. The server chooses *C* of the total clients *K* to participate throughout *T* communication rounds, hence selecting m=C×K clients every round. Every chosen client *c* gets the global model $G_{t}$ and conducts local training on its own dataset, hence generating an updated local model $L_{c}$. Sent back to the server, these local models are aggregated to update the global model via weighted average computation.

#### 2.3.2. FedAvg with VFL

VFL involves the collaboration of several clients or devices, where the data they possess are characterized by distinct and complimentary types or features [1]. Customers possess distinct characteristics or qualities of identical entities, which they divide vertically among themselves. We can express the VFL equation as follows:

(4) $${\mathrm{Model}}_{\mathrm{Global}}=f\left({\mathrm{Model}}_{{\mathrm{Local}}_{1}},\;{\mathrm{Model}}_{{\mathrm{Local}}_{2}},\;\ldots ,\;{\mathrm{Model}}_{{\mathrm{Local}}_{N}}\right)$$

where

- ${\mathrm{Model}}_{\mathrm{Global}}$ represents the global model trained across all clients.
- ${\mathrm{Model}}_{{\mathrm{Local}}_{i}}$ represents the local model trained on client *i*.
- $f({\mathrm{Model}}_{{\mathrm{Local}}_{1}\ldots })$ represents the function used to combine the local models into the global model. This function can vary based on the specific FL approach and may involve techniques such as model averaging, gradient aggregation, or secure multiparty computation.

The implementation of the FedAvg algorithm for VFL is different from when it is implemented in HFL, where data partitioning and communication patterns are different. In the VFL, data are divided such that individual samples fall into groups according to sets of the dataset that they belong to instead of falling into a category of individual samples. Each client has samples with different qualities of an identical set of the dataset. This brings further complexity to the table with communication and synchronization that allow to integrate data sources, yielding a more robust model. Algorithm 2 depicts the implementation of VFL, where each client initializes its local model with a feature set represented as $\theta_{i}$ for client *i*. The method runs across *T* communication rounds [48]. In each cycle, clients securely share intermediate results computed using their local models on their particular features with other clients. This ensures that model training incorporates the combined knowledge of all feature sets without sharing the actual data. For each client *i*, the local model is updated using gradient descent [49]. The local update rule is shown in Equation (5):

(5) $$\theta_{i}\leftarrow \theta_{i}-\eta \nabla L\left(\theta_{i}\right)$$

where η is the learning rate, and $L(\theta_{i})$ represents the local loss function. These local updates are performed for a specified number of local epochs *E*.
**Algorithm 2** Fed Average with VFL.**Require:** Total clients *K*, communication rounds *T*, local epochs *E*, learning rate η  1:**Initialization:**  2:Each client *i* initializes its local model $\theta_{i}$  3:**for** each round t=1, 2, …, T **do**  4:     **Client-side:**  5:     **for** each client i=1, 2, …, K **do**  6:          Compute intermediate results using local model $\theta_{i}$  7:          Securely share intermediate results with other clients  8:     **end for**  9:     **Server-side:**10:     **for** each client i=1, 2, …, K **do**11:          Receive intermediate results from other clients12:          Compute local updates based on shared results13:          Update local model $\theta_{i}$ using gradient descent:14:          $\theta_{i}\leftarrow \theta_{i}-\eta \nabla L\left(\theta_{i}\right)$15:     **end for**16:**end for**17:Combine local models to form global model through Equation (6)

After several communication rounds, the clients combine their locally updated models to form the global model. This can be expressed as

(6) $${\mathrm{Model}}_{\mathrm{Global}}=f\left(\theta_{1},\;\theta_{2},\;\ldots ,\;\theta_{K}\right)$$

where $\theta_{1},\;\theta_{2},\;\ldots ,\;\theta_{K}$ are the local models from each client. The function *f* represents the method of combining these local models, ensuring that the global model reflects the integrated knowledge of all participating clients.

By means of cooperative training, this iterative process of local updates and safe intermediary result exchange helps the global model to be improved using complementing input from many entities while maintaining data privacy. Therefore, the efficacy of VFL is found in scenarios where multiple companies have different characteristics for the same collection of events, such as in the financial or healthcare sectors. Table 2 refers to a summary of the support for Federated Averaging in horizontal and vertical federated settings across various FL frameworks.

## 3. Logistic Regression

Logistic regression is a technique used for both binary and multiclass classification. It models the probability of a categorical outcome based on one or more predictor variables, which can be either continuous or categorical. Logistic regression predicts the probability of a categorical dependent variable by fitting data to a logistic (logit) function [50]. For binary classification, the formula is shown in Equation (7)

(7) $$P\left(y=1|x\right)=\sigma \left(w^{T}x+b\right)$$

where

- P(y=1|x) is the probability that the dependent variable *y* equals 1 given the predictor variables *x*.
- $\sigma \left(z\right)=\frac{1}{1+e^{-z}}$ is the logistic function (also known as the sigmoid function).
- *w* is the vector of coefficients (weights).
- *x* is the vector of predictor variables.
- *b* is the intercept term (bias).

The logistic function σ(z) provides the guarantee that outputs are limited to the open interval (0, 1), which is suitable when modeling probabilities.

Logistic regression can be extended to multiclass classification by the use of a softmax function [51]. The softmax function is defined as shown in Equation (8)

(8) $$P\left(y=i|x\right)=\frac{e^{w_{i}^{T}x+b_{i}}}{\sum_{j=1}^{k}e^{w_{j}^{T}x+b_{j}}}$$

where

- P(y=i|x) is the probability that the dependent variable *y* equals class *i* given the predictor variables *x*.
- *k* is the number of classes.
- $w_{i}$ is the vector of coefficients for class *i*.
- $b_{i}$ is the intercept term for class *i*.

### 3.1. Logistic Regression Using FedAvg with HFL and VFL Setup

**Figure 5:**
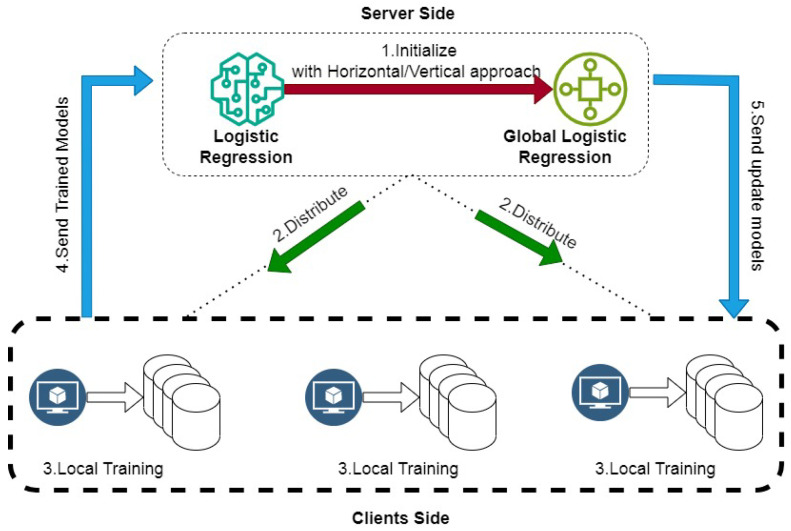
Logistic regression using FedAvg in HFL.

The implementation of logistic regression using FedAvg in a both VFL and HFL setup involves several steps for privacy-preserving and efficient model training across distributed clients (as depicted in Figure 5). The detailed steps are as follows.

#### 3.1.1. Initialize the Global Model

The central server initializes the global logistic regression model with horizontal or vertical setting parameters for the HFL or VFL setting parameter setup (data are distributed by samples), $w_{0}$ and $b_{0}$, where $w_{0}$ represents the initial weights and $b_{0}$ represents the initial bias [52]. These initial values are denoted as Equation (9)

(9) $$\beta_{0}^{\left(0\right)},\;\beta_{1}^{\left(0\right)},\;\ldots ,\;\beta_{n}^{\left(0\right)}$$

These initial values can be set to zero or small random values. For the VFL setup (data are distributed by features) according to Equation (10),

(10) $$W_{0}=\left[w_{0}^{\left(1\right)},\;w_{0}^{\left(2\right)},\;\ldots ,\;w_{0}^{\left(m\right)}\right]$$

where $w_{0}^{\left(i\right)}$ represents the initial weights for the *i*-th feature partition across *m* feature-partitioned clients.

#### 3.1.2. Distribute the Model to Clients

The central server distributes the initial model parameters $\beta_{0}^{\left(0\right)},\;\beta_{1}^{\left(0\right)},\;\ldots ,\;\beta_{n}^{\left(0\right)}$ to all participating clients.

#### 3.1.3. Local Training at Client Level

Each client performs the following steps:

- Receive the global model parameters;
- Update the model parameters on the local data for a few epochs. The update rule for logistic regression is based on gradient descent as shown in Equations (11) and (12)
  (11) $$w_{k}^{t+1}=w_{t}-\eta \nabla \ell_{k}\left(w_{t},b_{t}\right)$$
  (12) $$b_{k}^{t+1}=b_{t}-\eta \nabla \ell_{k}\left(w_{t},b_{t}\right)$$
  where η is the learning rate, and $\ell_{k}\left(w,b\right)$ is the local loss function at client *k*.

#### 3.1.4. Send Local Updates to Server

Each client sends its updated model parameters $w_{k}^{t+1}$ and $b_{k}^{t+1}$ to the central server.

#### 3.1.5. Aggregate Updates at Server Level

It is then based on these updates from all clients that the central server aggregates to form the new global model parameters. Usually, aggregation is done through the following averaging of the client updates, as shown in Equations (13) and (14):

(13) $$w_{t+1}=\frac{1}{K}\sum_{k=1}^{K}w_{k}^{t+1}$$

(14) $$b_{t+1}=\frac{1}{K}\sum_{k=1}^{K}b_{k}^{t+1}$$

where (K) is the number of clients.

#### 3.1.6. Repeat Until Convergence

Steps 3 to 5 are repeated for a number of rounds until the global model parameters converge to satisfaction.

## 4. Experiment and Results

This work implements different approaches of FL framework such as Flower, Flute, FedML, and PySyft running on centralized and decentralized FL topology, as shown in Table 3. We evaluated these simulation frameworks in terms of accuracy, computational time, and space. We utilized the FedAvg algorithm as the global model data partitioning technique in the logistic regression mode. Additionally, we assessed the space and time complexity of both HFL and VFL.

### 4.1. Exploratory Data Analysis (EDA)

The first step in performing this experiment involved an extensive exploratory data analysis (EDA) of the datasets used: MNIST [53] and Fashion-MNIST [54]. In the following section, we are going to discuss the exploratory data analysis steps. Initially, we began by loading the datasets and examining their shapes to ensure that they contain the expected number of samples and features. Then, we visualized sample images to get a sense of the data’s appearance.

#### 4.1.1. MNIST Dataset

**Figure 6:**
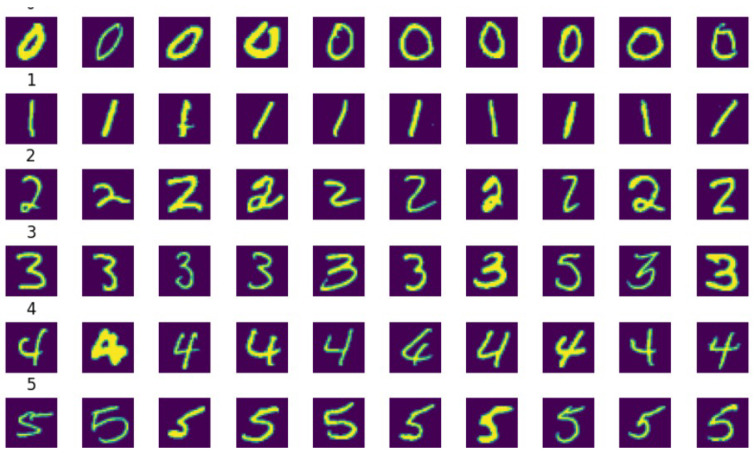
MNIST dataset representation.

**Figure 7:**
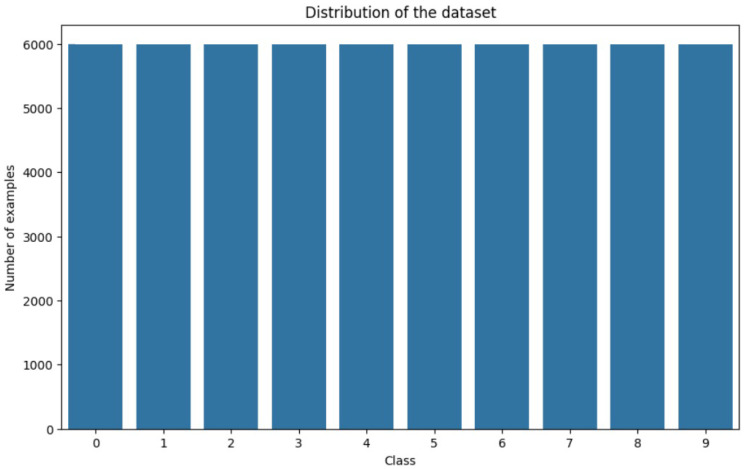
Class distribution of MNIST dataset.

Examining the MNIST dataset, we found that it comprised of 60,000 training images and 10,000 test images. Every picture highlighted a handwritten numeral between 0 and 9. Clear and distinguishable numerals displayed in a grayscale fashion with dimensions of 28 × 28 defined most of the visible example photos (as depicted in Figure 6). Due to its simplicity, this dataset was perfect for applications involving number recognition. The class distribution graph (as depicted in Figure 7) demonstrates an equal sample count in every digit class, indicating a well-balanced dataset. This balance ensures that the model shows no bias towards any specific number, thus guaranteeing satisfactory performance across all categories.

#### 4.1.2. Fashion-MNIST Dataset

**Figure 8:**
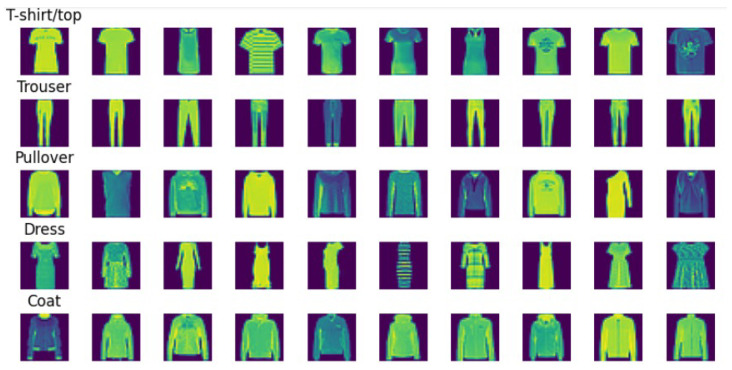
Fashion MNIST dataset representation.

**Figure 9:**
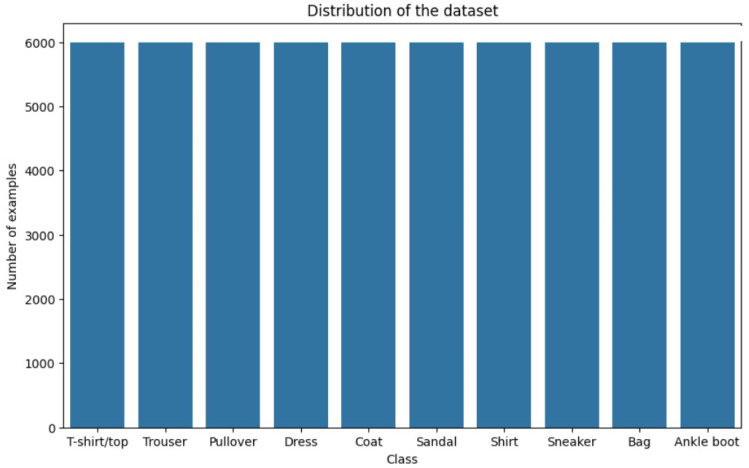
Class distribution of Fashion-MNIST dataset.

Comprising 10,000 test images and 60,000 training images, the Fashion MNIST dataset was examined. The pictures feature a variety of clothing, from T-shirts to jeans, sweaters, and skirts. Unlike the more simple MNIST figures, the graphic depiction of the example images highlighted the varied and complex character of the dataset. Though more sophisticated, the visuals still follow a 28 × 28 grayscale pattern, much like MNIST (as depicted in Figure 8). Fashion MNIST’s class distribution graph showed a fair mix from every class (as depicted in Figure 9).

### 4.2. Time Complexity of FL Framework

The time complexity of an FL framework, which refers to the computational cost of training models locally on the client device, depends on a few factors [55], which are going to be discussed further:

1. Local computation time (L): the duration of time required for a single device to finish a single iteration, during which it updates the local model. It mostly depends on model complexity and amount of data.
2. Communication time (C): depends on network bandwidth, the size of model updates, and the number of rounds of communication, i.e., cycles of model updates between the devices and the central server.
3. Synchronization time (S): in general, the time it takes to synchronize the updates from the different devices can be computationally determined given the heterogeneity in the devices, such as the computation power or speed of the network.
4. Aggregation time (A): the time required for the central server to aggregate the model updates received from devices. The aggregation involves simple calculations, such as an average.
5. Number of communication rounds (R): rounds of communication to reach the accuracy level of the model. It quantifies the total number of rounds of communication required for the model to reach a certain level of accuracy. It depends on factors such as the learning rate at which the model converges and the variability across devices.

The overall time complexity (*T*) of an FL framework can be expressed as a compound expression of these factors:

(15) T=R×(L+C+S+A)

What follows is the time complexity setting for both the VFL and HFL setups.

#### 4.2.1. Time Complexity Setting for HFL Setup


$$\begin{array}{cc} L_{HFL} & :\mathrm{Local}\;\mathrm{computation}\;\mathrm{time}\;\mathrm{on}\;a\;\mathrm{device}\;\mathrm{with}\;M\;\mathrm{samples}\;\mathrm{and}\;N\;\mathrm{parameters}\\ \; & \;\approx O(N\times M)\\ C_{HFL} & :\mathrm{Communication}\;\mathrm{time}\;\mathrm{for}\;K\;\mathrm{devices}\;\mathrm{and}\;N\;\mathrm{parameters}\\ \; & \;\approx O(K\times N)\\ S_{HFL} & :\mathrm{Synchronization}\;\mathrm{time}\;(\mathrm{varies},\;\mathrm{often}\;\mathrm{negligible})\\ A_{HFL} & :\mathrm{Aggregation}\;\mathrm{time}\;\mathrm{for}\;K\;\mathrm{devices}\\ \; & \;\approx O(K\times N)\\ R_{HFL} & :\mathrm{Number}\;\mathrm{of}\;\mathrm{communication}\;\mathrm{rounds}\;(\mathrm{depends}\;\mathrm{on}\;\mathrm{convergence}) \end{array}$$

#### 4.2.2. Time Complexity Setting for VFL Setup


$$\begin{array}{cc} L_{VFL} & :\mathrm{Local}\;\mathrm{computation}\;\mathrm{time}\;\mathrm{on}\;a\;\mathrm{device}\;\mathrm{with}\;\mathrm{shared}\;M\;\mathrm{samples}\;\mathrm{and}\;\mathrm{local}\;N_{i}\mathrm{parameters}\\ \; & \;\approx O(N_{i}\times M)\\ C_{VFL} & :\mathrm{Communication}\;\mathrm{time}\;\mathrm{for}\;K\;\mathrm{devices}\;\mathrm{with}\;\sum_{i=1}^{K}N_{i}\;\mathrm{parameters}\\ \; & \;\approx O(\sum_{i=1}^{K}N_{i})\\ S_{VFL} & :\mathrm{Synchronization}\;\mathrm{time}\;(\mathrm{varies},\;\mathrm{often}\;\mathrm{negligible})\\ A_{VFL} & :\mathrm{Aggregation}\;\mathrm{time}\;(\mathrm{complex},\;\mathrm{depends}\;\mathrm{on}\;\mathrm{method})\\ R_{VFL} & :\mathrm{Number}\;\mathrm{of}\;\mathrm{communication}\;\mathrm{rounds}\;(\mathrm{depends}\;\mathrm{on}\;\mathrm{convergence}) \end{array}$$

### 4.3. Space Complexity of an FL Framework

The space complexity of an FL framework refers to the amount of memory required to train an FL model, with data remaining decentralized across multiple devices [56]. Space complexity depends on several factors, which are discussed below:

1. Local model storage (M): the memory required to store the local model parameters on each device. This depends on the number of parameters in the model.
2. Local data storage (D): the memory required to store the local dataset on the local device. This depends on the size of the dataset.
3. Gradient storage (G): the memory required to store gradients computed during local training. This is typically proportional to the number of parameters.
4. Update storage (U): the memory required to store updates to be sent to the central server. This is usually similar to gradient storage.
5. Aggregation storage (A): the memory required by the central server to store and aggregate model updates from all devices. This depends on the number of devices and the size of the received models.

The overall space complexity (S) of an FL framework can be expressed as a compound expression of these factors:

(16) S=M+D+G+U+A

Below are the space complexity settings for the HFL and VFL setups.

#### 4.3.1. Space Complexity Setting for HFL


$$\begin{array}{cc} M_{HFL} & :\mathrm{Local}\;\mathrm{model}\;\mathrm{storage}\;\mathrm{on}\;a\;\mathrm{device}\;\mathrm{with}\;N\;\mathrm{parameters}\\ \; & \;\approx O(N)\\ D_{HFL} & :\mathrm{Local}\;\mathrm{data}\;\mathrm{storage}\;\mathrm{for}\;M\;\mathrm{samples}\\ \; & \;\approx O(M)\\ G_{HFL} & :\mathrm{Gradient}\;\mathrm{storage}\;\mathrm{for}\;N\;\mathrm{parameters}\\ \; & \;\approx O(N)\\ U_{HFL} & :\mathrm{Update}\;\mathrm{storage}\;\mathrm{for}\;N\;\mathrm{parameters}\\ \; & \;\approx O(N)\\ A_{HFL} & :\mathrm{Aggregation}\;\mathrm{storage}\;\mathrm{for}\;K\;\mathrm{devices},\;\mathrm{each}\;\mathrm{with}\;N\;\mathrm{parameters}\\ \; & \;\approx O(K\times N) \end{array}$$

#### 4.3.2. Space Complexity Setting for VFL


$$\begin{array}{cc} M_{VFL} & :\mathrm{Local}\;\mathrm{model}\;\mathrm{storage}\;\mathrm{on}\;a\;\mathrm{device}\;\mathrm{with}\;\mathrm{local}\;N_{i}\;\mathrm{parameters}\\ \; & \;\approx O(N_{i})\\ D_{VFL} & :\mathrm{Local}\;\mathrm{data}\;\mathrm{storage}\;\mathrm{for}\;\mathrm{shared}\;M\;\mathrm{samples}\\ \; & \;\approx O(M)\\ G_{VFL} & :\mathrm{Gradient}\;\mathrm{storage}\;\mathrm{for}\;\mathrm{local}\;N_{i}\;\mathrm{parameters}\\ \; & \;\approx O(N_{i})\\ U_{VFL} & :\mathrm{Update}\;\mathrm{storage}\;\mathrm{for}\;\mathrm{local}\;N_{i}\;\mathrm{parameters}\\ \; & \;\approx O(N_{i})\\ A_{VFL} & :\mathrm{Aggregation}\;\mathrm{storage}\;\mathrm{for}\;K\;\mathrm{devices},\;\mathrm{each}\;\mathrm{with}\;\mathrm{local}\;N_{i}\;\mathrm{parameters}\\ \; & \;\approx O\left(\sum_{i=1}^{K}N_{i}\right) \end{array}$$

### 4.4. Assessment of the Model’s Performance

To assess the model’s performance, we considered true positive (TP), true negative (TN), false negative (FN), and false positive (FP). Accuracy was calculated using the following equation:

(17) Accuracy=(TP+TN)/(TP+TN+FP+FN),

where

- True positives (TPs): the prediction is “yes” and the actual result is “yes”.
- True negatives (TNs): the prediction is “no” and the actual result is “no”.
- False negatives (FNs): the prediction is “no” and the actual result is “yes”.
- False positives (FPs): the prediction is “yes” and the actual result is “no”.

### 4.5. Hardware Setup

The experiments were conducted using Google Colab, a cloud-based platform that provides access to powerful hardware and is widely used for machine learning research. The specific setup was as follows:

- Processor: 2 x Intel(R) Xeon(R) CPU @ 2.20 GHz (virtual CPUs);
- Memory: 13 GB RAM;
- GPU: NVIDIA Tesla T4 or P100 (dependent on session availability), each with 16 GB VRAM; storage: approximately 50 GB of disk space available in each Colab session.

### 4.6. Software Setup

The software environment was set up using Python and relevant libraries to ensure the compatibility and performance of the FL frameworks. The details are as follows:

- Operating system: Debian-based environment provided by Google Colab;
- Python version: 3.7;
- FL frameworks and libraries:
  – FedML: installed via pip install fedml, version 0.7.1;
  – Flower: installed via pip install flwr, version 0.18.0;
  – Flute: custom setup and integration on the version released on 14 August 2023;
  – GitHub repository to match the Colab environment Syft: installed via pip install syft, version 0.2.9;
  – FedScale: installed via pip install fedscale, version 0.1.2.

### 4.7. Experimental Setting and Results for MNIST Dataset

Using the four different open frameworks of FL, Flower, Flute, FedML, and PySyft, the current experiment on the MNIST dataset aimed to study the accuracy of a logistic regression model as well as the time and space complexity in FL models using the FedAvg algorithm. Both Flower and Flute were configured on the HFL approach, while FedML and PySyft were configured on the VFL approach. The number of users, local epochs, and global epochs varied across the settings. Table 4 summarizes the accuracy for different settings.

**Figure 10:**
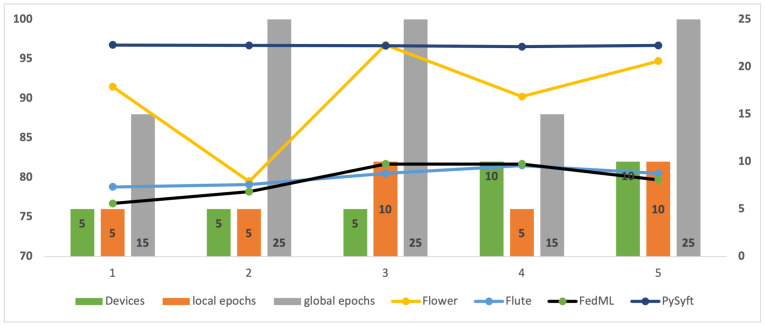
The performance of the simulators on the MNIST dataset.

Overall (as depicted in Figure 10), Flower has overcome both FedML and Flute in term of accuracy in several testing scenarios. For instance, for five users with five local epochs and fifteen global epochs, Flower reaches an accuracy of 91.48%, while FedML and Flute reach accuracy of 76.7% and 78.8%, respectively. The increased number of local epochs improve the performance of the model. Notably, the accuracy reaches 95.78% as Flower’s local epochs are increased to 10 and global epochs to 25. With an increase in the number of global epochs and users to 25 and 10, respectively, Flower attains an accuracy of 94.72%. In contrast, for the same configuration, Flute achieves an accuracy of 80.5%, while FedML achieves an accuracy of 79.7%. While PySyft performs very well in most cases, it does not consistently reach the highest accuracy levels of Flower. It records an accuracy of 96.66% with five users, 10 local epochs, and 25 global epochs. These results clearly depict the efficacy of Flower in achieving higher accuracy in federated settings.

### 4.8. Experimental Setting and Results for Fashion MNIST

The next experiment was carried out on the Fashion MNIST dataset to examine the accuracy of FL models by using the same hardware and software configuration. The number of users, local epochs, and global epochs all differed in the experimental setups. Table 5 compiles the accuracy outcomes for various settings.

**Figure 11:**
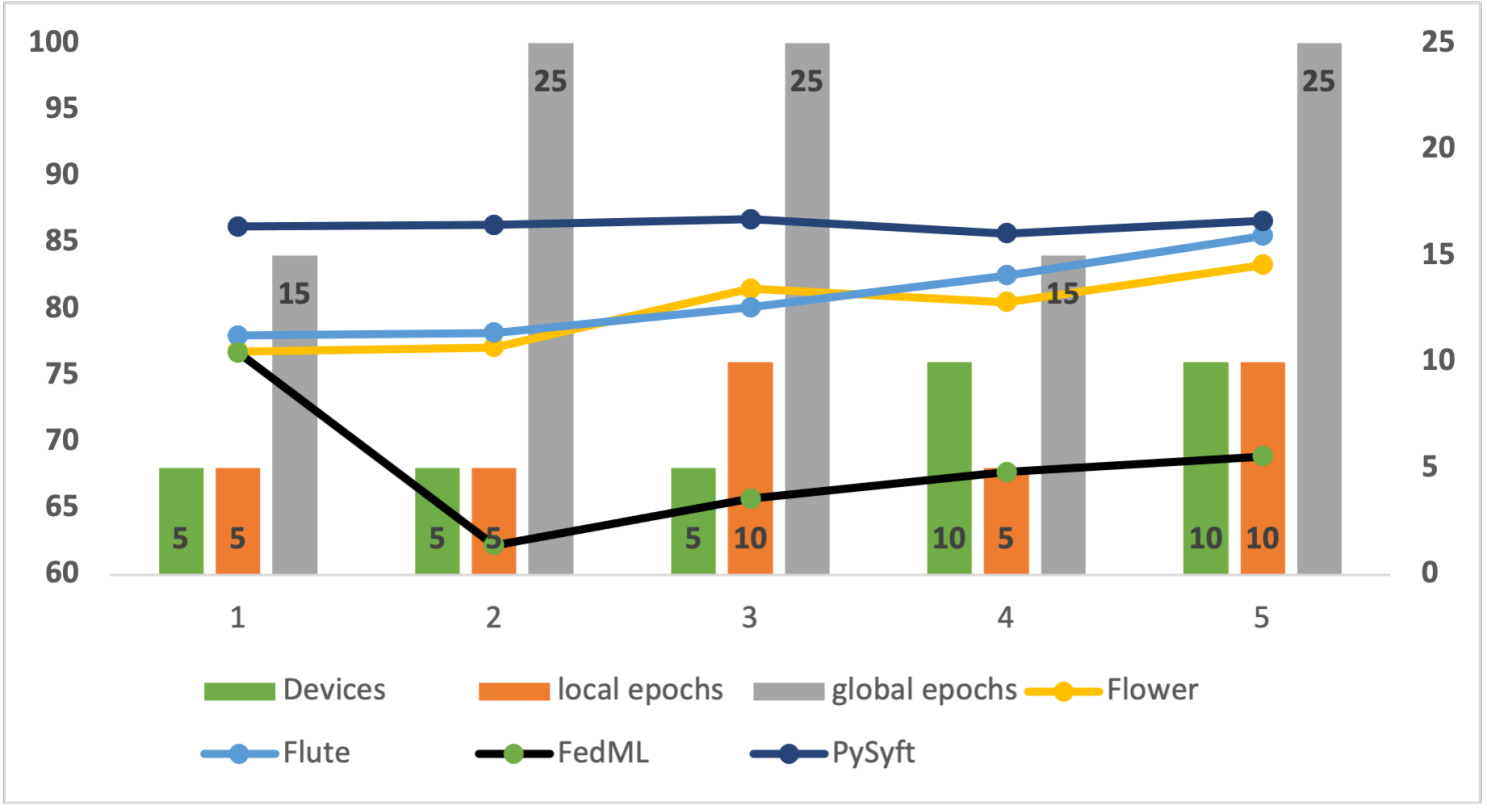
The performance of the simulators on the Fashion-MNIST dataset.

The experimental analysis of the Fashion MNIST dataset using the Flower, Flute, FedML, and PySyft simulators reveals that the PySyft simulator maintains the highest accuracy among configurations (as depicted in Figure 11). For example, the configuration with five users, five local epochs, and fifteen global epochs achieves an accuracy of 86.18% for PySyft. In comparison, Flower achieves an accuracy of 76.8%, Flute achieves an accuracy of 78%, and FedML achieves an accuracy of 76.7%. Even when increasing local epochs to 10 and global epochs to 25, PySyft achieves an accuracy of 86.73%. These results indicate that PySyft is robust and powerful in FL tasks on the Fashion MNIST dataset.

## 5. Time and Space Complexity Analysis of FL Frameworks

The time and space complexity of FL frameworks are bound to vary depending on the dataset and how the learning process is configured. In this section, we have analyzed the time and space complexity for the Flower, Flute, FedML, and PySyft frameworks on both the MNIST and Fashion MNIST datasets.

- N: number of parameters in the model.
- M: number of samples in the local dataset.
- N_i: number of local parameters in the model for device *i*.
- K: number of devices (clients).
- R_HFL: number of communication rounds for HFL.
- R_VFL: number of communication rounds for VFL.
- negligible: indicates that synchronization time is often negligible.
- complex: indicates that aggregation time for VFL can be complex and method-dependent.

The time and space complexity of both the MNIST and Fashion MNIST datasets depend on many factors: number of model parameters, number of local samples, number of devices, and number of rounds of communication.

The time complexity, denoted as $T_{HFL}$ and $T_{VFL}$, describes the computational efforts towards the training and communication in HFL and VFL settings, respectively. At the same time, the space complexity refers to the memory requirements for model parameter and data storage, and the intermediate results in the process. For HFL, both Flower and Flute have a time and space complexity that are the same, dominated by operations O(N×M) and O(K×N) for the local training and aggregation processes, respectively. The space complexity includes terms for model parameters, data samples, and aggregated results. In contrast, our analysis reveals that for FedML and PySyft, evaluated in the context of VFL, time complexity includes terms such as $O(N_{i}\times M)$ for local training and $O(\sum_{i=1}^{K}N_{i})$ for aggregation, which become complex. Additionally, space complexity accounts for local parameters and aggregated results, scaling linearly with the number of clients *K*. It means that the time and space complexity for the MNIST and Fashion MNIST datasets would be similar in value, as they have images of approximately the same size and structure. Specific implementation and optimization in every framework could bring about differences in real performance. For instance, the actual computation times and memory usage, as shown in Table 6, highlight that Flute has the shortest computation time and lowest memory usage, while PySyft, despite its high accuracy, has longer computation times. This comprehensive analysis underscores the trade-offs between accuracy, computation time, and memory usage in FL frameworks, guiding the selection of appropriate frameworks for different application scenarios.

## 6. Results for CIFAR-100 Dataset

In this section, we extend our analysis to the CIFAR-100 dataset, which is rather challenging due to the large number of classes and very fine details of the images. The CIFAR-100 dataset contains 100 classes, each containing 600 images. In total, there are 500 training images and 100 testing images per class with a resolution of 32 × 32 pixels. This dataset presents a more challenging case for FL models as compared to MNIST and Fashion-MNIST, attributed to the detailed granularity in the data [57].

### Experimental Setup

In our experiments, we focused on two leading frameworks: FedML and Flower. We used logistic regression, since it is efficient for most data types while taking into our account VFL and HFL. The evaluation was based on three key metrics: accuracy, F1-score, and training time. Accuracy estimates the proportion of correctly predicted instances. The F1-score is a balanced measure of precision and recall, and it practically helps in the evaluation of class performance. The training time has an important role in practical deployment: it gives how well a framework is geared for efficient training. The results, as summarized in Table 7, show that FedML generally outperforms Flower across various numbers of clients, specifically in accuracy and F1-score. For instance, with five clients, FedML achieves an accuracy of 68% and an F1-score of 67%, slightly higher than Flower’s 67% accuracy and 67% F1-score. As the number of clients increases, both frameworks show a decline in performance, likely due to the challenges associated with distributing and aggregating data across more nodes. With 10 clients, FedML maintains its lead with an accuracy of 67% and an F1-score of 66%, compared to Flower’s 66% accuracy and 65% F1-score. This trend continues with 50 and 100 clients, where FedML records accuracies of 58% and 54%, respectively, and corresponding F1-scores of 58% and 53%. Flower’s performance metrics slightly lag behind, with accuracies of 57% and 51% and F1-scores matching these accuracy values. These results highlight the robustness of FedML in handling larger federations of clients, possibly due to its optimized data aggregation and processing capabilities.

The training times for the Flower and FedML frameworks, as shown in Table 8, reveal significant differences in efficiency between the two. The table presents the training duration in seconds for both frameworks across different numbers of clients, specifically 5, 10, 50, and 100. This analysis is crucial for understanding the computational efficiency of each framework, especially in large-scale FL setups where time and resource optimization are vital.For a setup with five clients, Flower requires a significantly longer training time of 2500 s compared to FedML’s 500 s. This stark difference indicates that FedML’s architecture is more optimized for quicker data processing and model updates, possibly due to more efficient communication protocols or model aggregation techniques. As the number of clients increases, the training time for Flower also rises markedly, reaching 2750 s for 10 clients, 3000 s for 50 clients, and 3500 s for 100 clients. In contrast, FedML maintains a consistent training time of 500 s for up to 50 clients, only increasing slightly to 600 s with 100 clients. This suggests that FedML handles scaling more efficiently, likely due to better parallelization and reduced communication overhead. The consistent increase in training time for Flower as the number of clients increases indicates potential bottlenecks in its architecture, such as less efficient data handling or synchronization mechanisms. These bottlenecks can lead to higher latency and slower convergence rates, particularly in scenarios involving a large number of clients. On the other hand, FedML’s more stable training times highlight its ability to manage large federations of clients without significant degradation in performance, making it a more scalable option for practical FL applications.

## 7. Our Findings

In this section, we present the key findings from our comprehensive study on FL systems. Our research aimed to dissect the nuances of various FL topologies and frameworks, providing insights into their strengths, weaknesses, and practical applications. The findings are categorized into three main areas: theoretical analysis, empirical evaluation, and practical implications. These categories help distinguish the different aspects of our contribution and provide a clear understanding of the distinct elements explored in our study. Below, we detail each category, emphasizing the unique contributions and insights gained:

- Analysis of advantages and drawbacks: this contribution focuses on a theoretical examination of decentralized and centralized FL topologies. We provide a detailed discussion on the inherent benefits and limitations of each approach, with a particular emphasis on privacy, scalability, and robustness which summarized in Table 9.
- Evaluation of FL frameworks: this distinct contribution involves the practical assessment of existing FL frameworks, including Flower, Flute, FedML, and PySyft. We conducted empirical evaluations using logistic regression with the FedAvg algorithm across both HFL and VFL approaches, offering a comparative analysis based on performance metrics and use-case suitability.
- Implications and recommendations: we further contribute by discussing the implications of different data distribution strategies and providing actionable recommendations for enhancing the effectiveness of FL systems. This includes insights into choosing appropriate simulation frameworks for specific research and practical applications.

## 8. Lessons Learned

The experimental evaluation and the corresponding analysis of the complexity in FL frameworks applied to both datasets demonstrated several valuable lessons. These insights will guide future research and practical implementations of FL in various scenarios.

### 8.1. Selection of Framework and Accuracy

The framework choices for FL is always one of the main characteristics in identifying the accuracy in the generated models. In our experiments, Flower was found to be very close to the highest accuracy in almost all settings for both the MNIST and Fashion MNIST datasets, ahead of several prior frameworks. PySyft also turned in a strong performance, in cases with the FMNIST dataset. Therefore, choosing the appropriate framework in view of the exact dataset and requirements of applications is of much importance in attaining optimal performance.

### 8.2. Impact of Training Configurations

The number of users, local epochs, and global epochs are the most critical factors in any FL model. In the experiments with Flower and PySyft, the models’ accuracy tended to increase with an increased number of local epochs. The impact of these parameters may differ across different frameworks, thus showing a fine-tuning requirement of training configurations with respect to a trade-off between model accuracy and computational efficiency.

### 8.3. Time and Space Complexity

FL frameworks are substantially dependent on knowledge about time and space complexity, which enable the efficient utilization of resources. Based on our analysis, it is evident that both Flower and Flute, as HFL setups, have similar time and space complexity. However, FedML and PySyft are the studied VFL setups that expressed more complicated and method-dependent aggregation times. In the comprehensive evaluation, this was depicted by Flute having the shortest computation time and lowest memory usage, while, in contrast, PySyft required the longest computation times to attain very high accuracy. Hence, these results further reiterate the need to consider computational and memory requirements together in picking an FL framework.

### 8.4. Dataset Characteristics and Model Performance

The performance of FL models is driven by the characteristics of the dataset, notably in terms of size and complexity. While the MNIST and Fashion MNIST datasets are generally of comparable size and structure, it has been indicated that additional challenges were created by the more complex nature of Fashion MNIST images for accurate model training. This puts a high demand on customized approaches and framework optimizations in dealing with divergent data sets.

## 9. Conclusions and Future Work

In this paper, we discussed and examined the characteristics of centralized and decentralized FL topology. Centralized FL, characterized by its simpler topology and higher effectiveness, has been more thoroughly analyzed and extensively deployed in real-world applications. In contrast, a decentralized FL topology, which directly intercommunicates the devices and then aggregates the models locally, is an approach motivated by the high communication costs and challenges associated with configuring all participating clients. We performed benchmarking on a few FL simulators, among which were Flower, Flute, FedML, and PySyft, in the search for an optimal set of configurations that would give the maximum accuracy at a given point. The benchmarking was performed on the MNIST and Fashion-MNIST datasets, using a logistic regression model aggregated with the FedAvg algorithm on both HFL and VFL. Our results show huge variance in model performance and accuracy for different simulators on the same topology. This points out the fact that the right choice of simulator is important for the specific applications and calls for customized solutions tailored to the specific requirements of each use case. In future work, we plan to extend our analysis by experimenting with a broader array of datasets using various simulators and further segmenting data into smaller, more homogeneous groups to enhance model accuracy. Additionally, we will incorporate experiments that utilize both decentralized and hybrid FL topologies to explore their performance. In conclusion, a detailed comparison of various frameworks in this paper contributes to advancing FL and delineates the trade-offs between centralization and decentralization. The findings in terms of critically important aspects, among others, are the FL topology and communication efficiency within FL systems that, at the same time, pave the way for more advanced practical solutions in FL.

## Figures and Tables

**Table 1:** Summary of solutions and limitations of various papers.

Paper	Solution	Limitations
McMahan et al. (2016) [1]	Introduced FL to enhance conventional machine learning (ML) by training datasets from multiple sources in a decentralized manner, thus preserving data privacy.	This work primarily focused on the concept rather than on practical applications, and did not address scalability issues extensively.
Bonawitz et al. [6]	Implemented one of the first FL models using mobile devices, allowing devices to train and process local data before sharing the model with a central server.	The approach still relies on a centralized server for model aggregation, and the initial implementation had limited real-world application scenarios.
Mohamed et al. [10]	Developed a Federated Deep Learning-based Intrusion Detection System (FED-IDS) for smart transportation systems, using vehicular edge computing (VEC) and blockchain for local update verification.	The solution is limited to vehicular networks and transportation systems, primarily focusing on security and intrusion detection.
Whitworth et al. [11]	Proposed a hybrid approach for detecting DDoS attacks using a multi-channel CNN-GRU methodology, achieving high accuracy through a comprehensive four-step process.	The method is limited to specific datasets and scenarios, focusing solely on DDoS attacks rather than other types of cyber threats.
Koroniotis et al. [12]	Provided a review of IoT integration in smart airports, discussing the benefits and vulnerabilities of IoT in airport services.	The paper is mainly a review and does not introduce new methods or models, with only a brief discussion of vulnerabilities.
Wu et al. [13]	Compared FL frameworks like Flower, LEAF, Syft, and FedScale, identifying Flower’s strengths in communication-agnostic and language-agnostic capabilities.	The study focused on specific frameworks without including newer or lesser-known ones, and scalability was mainly tested in simulated environments.
Hipolito et al. [14]	Evaluated FL frameworks FedML, Flower, and Flute, finding that Flute supports performance optimizations and integration with both cloud and multi-GPU environments.	Found that Flower lacks support for cloud integration and multi-GPU environments, with practical deployment issues in real-world scenarios not addressed.
Our Work	Analyzed different FL simulators and compared four of them using two different datasets, focusing on both VFL and HFL.	Focused on evaluating existing frameworks without proposing a new FL framework.

**Table 2:** Support for FedAvg approach in HFL and VFL.

Paper Reference	Framework Name	Horizontal Support	Vertical Support
[14]	Flute	✓	×
[41]	Flower	✓	×
[8]	FedML	✓	✓
[40]	PySyft	✓	✓

**Table 3:** Comparison of FL simulators.

Simulator	Centralized Learning	Decentralized Learning
Flower	✓	×
Flute	✓	×
FedML	✓	✓
PySyft	✓	✓

**Table 4:** Models’ accuracy on MNIST dataset.

No. of Users	Local Epochs	Global Epochs	Flower	Flute	FedML	PySyft
5	5	15	91.48	78.8	76.7	96.76
5	5	25	79.5	79.1	78.2	96.71
5	10	25	95.78	80.5	81.7	96.66
10	5	15	90.23	81.5	81.7	96.55
10	10	25	94.72	80.5	79.7	96.7

**Table 5:** The performance of simulators on Fashion-MNIST dataset using different settings.

No. of Users	Local Epochs	Global Epochs	Flower	Flute	FEDML	PySyft
5	5	15	76.8	78	76.7	86.18
5	5	25	77.1	78.2	62.2	86.31
5	10	25	81.5	80.1	65.7	86.73
10	5	15	80.5	82.5	67.7	85.68
10	10	25	83.3	85.5	68.9	86.62

**Table 6:** Time and space complexity for Flower, Flute, FedML, and PySyft in HFL and VFL setups.

Framework	Time Complexity (T)	Space Complexity (S)
Flower	$T_{HFL}=R_{HFL}\times \left(O\left(N\times M\right)+O\left(K\times N\right)+\mathrm{negligible}+O\left(K\times N\right)\right)$	$S_{HFL}=O\left(N\right)+O\left(M\right)+O\left(N\right)+O\left(N\right)+O\left(K\times N\right)$
Flute	$T_{HFL}=R_{HFL}\times \left(O\left(N\times M\right)+O\left(K\times N\right)+\mathrm{negligible}+O\left(K\times N\right)\right)$	$S_{HFL}=O\left(N\right)+O\left(M\right)+O\left(N\right)+O\left(N\right)+O\left(K\times N\right)$
FedML	$T_{VFL}=R_{VFL}\times \left(O\left(N_{i}\times M\right)+O\left(\sum_{i=1}^{K}N_{i}\right)+\mathrm{negligible}+\mathrm{complex}\right)$	$S_{VFL}=O\left(N_{i}\right)+O\left(M\right)+O\left(N_{i}\right)+O\left(N_{i}\right)+O\left(\sum_{i=1}^{K}N_{i}\right)$
PySyft	$T_{VFL}=R_{VFL}\times \left(O\left(N_{i}\times M\right)+O\left(\sum_{i=1}^{K}N_{i}\right)+\mathrm{negligible}+\mathrm{complex}\right)$	$S_{VFL}=O\left(N_{i}\right)+O\left(M\right)+O\left(N_{i}\right)+O\left(N_{i}\right)+O\left(\sum_{i=1}^{K}N_{i}\right)$

**Table 7:** Performance metrics for Flower and FedML frameworks with CIFAR-100.

Framework	Metric	Clients = 5	Clients = 10	Clients = 50	Clients = 100
Flower	Accuracy	67	66	57	51
	F1 Score	67	65	57	51
FedML	Accuracy	68	67	58	54
	F1 Score	67	66	58	53

**Table 8:** Training time (in seconds) for Flower and FedML frameworks with different numbers of clients.

Clients	Simulators	Flower	FedML
5	Training Time (seconds)	2500	500
10	Training Time (seconds)	2750	500
50	Training Time (seconds)	3000	500
100	Training Time (seconds)	3500	600

**Table 9:** Advantages and disadvantages of each FL framework.

Framework	Advantages	Disadvantages
Flower	High accuracy in most settings for both MNIST and Fashion MNIST datasets. Consistent performance across various configurations.	None explicitly mentioned, but no standout issues were identified.
Flute	Shortest computation time. Lowest memory usage.	Lower accuracy compared to other frameworks, especially in more complex scenarios.
FedML	Capable of handling complex aggregation in VFL.	Higher complexity in aggregation processes. May require more resources for optimal performance.
PySyft	High accuracy, particularly noted with the FMNIST dataset.	Longest computation times. Higher resource consumption in terms of memory and processing time.

## Data Availability

The data used in this work are available on Kaggle.
